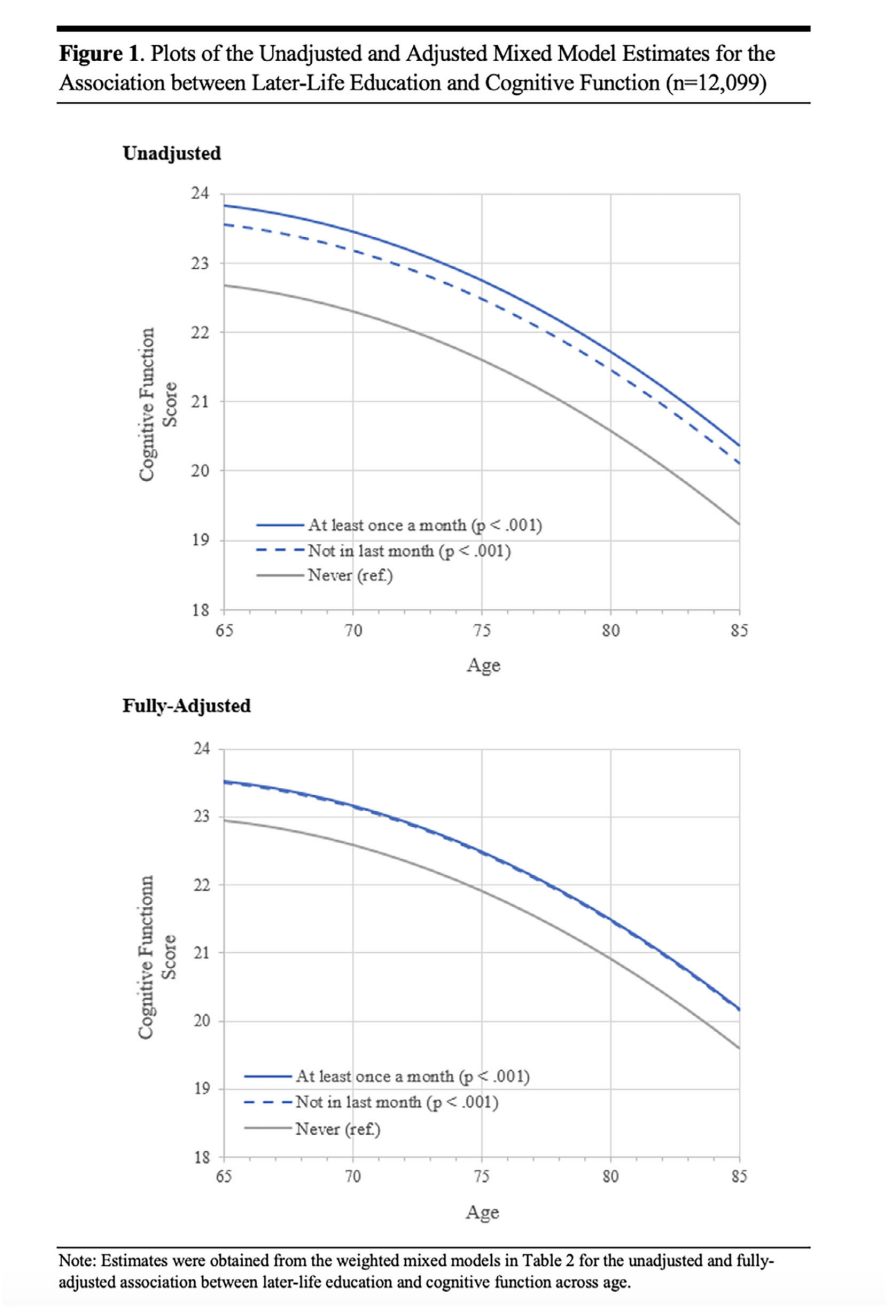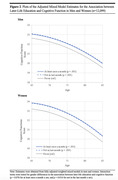# The Impact of Later‐Life Education on Trajectories of Cognitive Functionamong U.S. Older Adults

**DOI:** 10.1002/alz.086196

**Published:** 2025-01-09

**Authors:** Nan Wang, Radha Dhingra, Ying Xian, Eleanor S McConnell, Bei Wu, Matthew E. Dupre, Hanzhang Xu

**Affiliations:** ^1^ University of California Davis, Davis, CA USA; ^2^ Duke University, Durham, NC USA; ^3^ UT Southwestern Medical Center, Dallas, TX USA; ^4^ NYU Aging Incubator, New York, NY USA; ^5^ New York University, New York, NY USA; ^6^ Duke‐NUS Medical School, Singapore, Singapore Singapore

## Abstract

**Background:**

Little is known about how education in later life is related to cognitive function in older adults. This study assessed whether participating in later‐life education was associated with better cognitive function over time and whether the benefits differed by sex, race/ethnicity, and prior education level in a nationally‐representative sample of U.S. older adults.

**Method:**

We conducted a retrospective cohort study using six waves of data from the Health and Retirement Study ([HRS] 2008‐2018) that included adults aged ≥ 65 years with no baseline diagnosis of dementia. Cognitive function was measured at baseline and over time using a summary score (range = 0‐35). Later‐life education was measured at every wave and was categorized as attended an educational or training course “at least once a month or more” (10.3%), “not in the last month” (45.5%), or “never” (44.3%). Inverse probability weighting was applied to account for potential selection bias in who participated in later‐life education. The associations between later‐life education and trajectories of cognitive function were estimated using weighted mixed‐effects linear regression models.

**Result:**

Of the 12,099 participants (median [interquartile range] age, 71[67‐77]), engaging in any later‐life education, either at least once a month (0.56 points higher, 95% CI, 0.40 to 0.73; *P* < .001) or not in the last month (0.55 points higher, 95% CI, 0.45 to 0.65; *P* < .001) was associated with significantly better cognitive function over time compared to never participating in later‐life education. The association remained constant as people aged and was only partially attenuated after adjusting for sociodemographic, psychosocial, behavioral, and health‐related factors. In addition, the benefits of engaging in any later‐life education on cognitive function were greater in women than in men (at least once a month: 0.30 points higher, 95% CI, ‐0.03 to 0.63, *P* = .076, not in the last month: 0.24 points higher, 95% CI, 0.04 to 0.43, *P* = .016) but no significant differences by race/ethnicity or prior educational attainment.

**Conclusion:**

Engaging in later‐life education is beneficial for cognitive function and underscores the importance of continued learning among older adults.